# Insulin degludec improves health-related quality of life (SF-36®) compared with insulin glargine in people with Type 2 diabetes starting on basal insulin: a meta-analysis of phase 3a trials

**DOI:** 10.1111/dme.12086

**Published:** 2013-01-21

**Authors:** N Freemantle, L Meneghini, T Christensen, M L Wolden, J Jendle, R Ratner

**Affiliations:** 1 Department of Primary Care and Population HealthUCL Medical School, London, UK; 2 Division of Endocrinology and Diabetes, University of Miami Miller School of MedicineMiami, FL, USA; 3 Novo Nordisk A/SSøborg, Denmark; 4 School of Health and Medical Sciences, Örebro UniversityÖrebro, Sweden; 5 American Diabetes AssociationAlexandria, VA, USA

## Abstract

**Aim** To compare the effect of insulin degludec and insulin glargine on health-related quality of life in patients with Type 2 diabetes starting on insulin therapy.

**Methods** Patient-level data from three open-label, randomized, treat-to-target trials of 26 or 52 weeks' duration were pooled using a weighted analysis in conjunction with a fixed-effects model. Insulin-naive patients received either insulin degludec (*n* = 1290) or insulin glargine (*n* = 632) once daily, in combination with oral anti-diabetic drugs. Glycaemic control was assessed via HbA_1c_ and fasting plasma glucose concentrations. Rates of hypoglycaemia, defined as plasma glucose < 3.1 mmol/l (< 56 mg/dl), were recorded. Health-related quality of life was evaluated using the 36-item Short Form (SF-36®) version 2 questionnaire. Statistical analysis was performed using a generalized linear model with treatment, trial, anti-diabetic therapy at baseline, gender, region and age as explanatory variables.

**Results** Insulin degludec was confirmed as non-inferior to insulin glargine based on HbA_1c_ concentrations. In each trial comprising the meta-analysis, fasting plasma glucose and confirmed overall and nocturnal (00.01–05.59 h) hypoglycaemia were all numerically or significantly lower with insulin degludec vs. insulin glargine. At endpoint, the overall physical health component score was significantly higher (better) with insulin degludec vs. insulin glargine [+0.66 (95% CI 0.04–1.28)], largely attributable to a difference [+1.10 (95% CI 0.22–1.98)] in the bodily pain domain score. In the mental domains, vitality was significantly higher with insulin degludec vs. insulin glargine [+0.81 (95% CI 0.01–1.59)].

**Conclusions** Compared with insulin glargine, insulin degludec leads to improvements in both mental and physical health status for patients with Type 2 diabetes initiating insulin therapy.

## Introduction

Type 2 diabetes has been shown to have a negative impact on health-related quality of life 1. Diabetes complications are a major contributor to this deterioration in quality of life, with patients with diabetes experiencing higher rates of morbidity and mortality compared with the general population 2,3. The treatment of Type 2 diabetes with exogenous insulin significantly improves prognosis for patients, by restoring glycaemic control and consequently reducing morbidity 4. However, despite the clear advantages to health associated with good glycaemic control, insulin therapy does not completely eliminate the physical and psychosocial burden of diabetes. Fear of hypoglycaemia is commonly cited by both patients and physicians as a challenge in the initiation of, and adherence to, insulin treatment regimens 5. Other psychosocial concerns affecting patients with diabetes include fear of self-injection, anxiety from inflexible or complicated dosing regimens and embarrassment associated with diagnosis 6. There is also significant overlap between the physical and mental aspects of health-related quality of life in diabetes. Co-morbid depression has been associated with impaired self-management and worsening of metabolic control and, in return, deterioration in metabolic control can worsen mental health 7.

There is increasing recognition from the medical community and healthcare payers that health-related quality of life is an important component of diabetes management for individuals and society as a whole, and should be taken into account when evaluating the efficacy of new treatments.

Notwithstanding, assessment of health-related quality of life during clinical trials is uncommon, with only 14% of all trials between 2004 and 2007 recording patient-reported outcomes 8. Typically, assessments have used disease-specific measures, rather than more generic metrics, making any comparison across treatments, conditions and populations difficult, if not impossible 9. The 36-item Short Form (SF-36®) health questionnaire was designed to overcome these issues and has been successfully used to measure health outcomes in a variety of studies 10, becoming the most widely used instrument for measuring health-related quality of life in clinical trials 8. SF-36 scores have shown a strong association with a range of clinical anchors, from 2-year mortality risk and morbidity to unemployment 10.

Insulin degludec is a new-generation, ultra-long-acting basal insulin. Insulin degludec has a unique mode of protraction, whereby it forms soluble multi-hexamers upon subcutaneous injection. Insulin degludec monomers slowly and continuously dissociate from these multi-hexamers into the circulation, ensuring a flat and stable pharmacokinetic profile and a duration of action lasting > 42 h 11,12. In previous studies, insulin degludec exhibited lower rates of day-to-day and hour-to-hour variability in glucose-lowering activity compared with insulin glargine 13. The efficacy of insulin degludec is comparable with that of insulin glargine 14–17. Furthermore, at equivalent levels of glycaemic control, insulin degludec demonstrates a lower risk of hypoglycaemia compared with insulin glargine 16,18–20. Insulin degludec has previously shown significantly better health-related quality of life outcomes compared with insulin glargine (as measured with the SF-36 instrument) in patients with Type 1 diabetes 17 and in patients with Type 2 diabetes on a basal–bolus regimen 16.

The aim of this pre-planned meta-analysis was to compare the effect of insulin degludec and insulin glargine on health-related quality of life in patients with Type 2 diabetes starting on basal insulin, in combination with oral anti-diabetic drugs.

## Patients and methods

### Study design and selection criteria

This meta-analysis incorporated pooled, patient-level data from three phase 3a trials comparing insulin degludec once daily with insulin glargine once daily. All three clinical trials were randomized, controlled, open-label, multi-centre, confirmatory, treat-to-target interventions of 26 or 52 weeks' duration in patients with Type 2 diabetes 21–23. Subjects (insulin degludec *n* = 1290; insulin glargine *n* = 632) were enrolled into the trials if they were ≥ 18 years old (≥ 20 in Japan) and had been diagnosed with diabetes ≥ 6 months, with HbA_1c_ between 53 and 86 mmol/mol (7–10%) and a BMI ≤ 40 kg/m^2^. All subjects were insulin naive and were excluded if they had a history of recurrent severe hypoglycaemia (defined as > 1 event in the preceding 12 months). Insulin doses were titrated to a target fasting plasma glucose concentration of 5 mmol/l (90 mg/dl). Glycaemic control was assessed by measurement of HbA_1c_ and fasting plasma glucose. The rate of hypoglycaemic events was recorded in each trial as part of the safety analysis. Detailed descriptions of the study population and protocol are available in previously published studies 21–23.

### Quality-of-life (SF-36) assessment

Quality of life was evaluated using the validated SF-36 version 2 health survey 10. The SF-36 is a multi-purpose questionnaire, comprising 36 questions distributed across eight scales (see also Supporting Information, Fig. S1). Two summary measures—physical and mental health—are calculated from four scales each. Higher scores represent improved health status, with a score of 50 being the mean for the general population. The SF-36 is a generic measure, which means that it can be used to assess the impact of any disease, having previously been utilized in large-scale, population-based surveys 24. In the present study, the questionnaire was completed by trial patients at baseline and end of trial.

### Statistical analysis

Pooling of trials was performed using a weighted analysis in conjunction with a fixed-effects model. Missing values were imputed by means of last observation carried forward. Statistical evaluation of endpoints was carried out using analysis of variance (ANOVA) with treatment; anti-diabetic therapy at baseline; gender, trial and region as fixed factors; and age and baseline values (SF-36 scores) as covariates. SF-36 values are presented as least squares means ± standard error. The primary analysis of SF-36 scores in this investigation examined between-treatment differences. A supportive analysis of within-treatment differences was also conducted, to assess whether the treatment regimens were related to baseline values.

## Results

A total of 1922 patients were included in the present analysis. The subjects were representative for patients with Type 2 diabetes starting on basal insulin therapy, with disease duration of 9.5 years, HbA_1c_ above target [67 mmol/mol (8.3%)] and moderate obesity (BMI 25–33 kg/m^2^). Subject demographics and baseline characteristics are shown in Table 1.

**Table 1:** Baseline characteristics by trial and meta-analysis

	NN3579 BEGIN™ Once Long	NN3586 BEGIN™ Once Asia	NN3672 BEGIN™ Low Volume	Total
*n*	1030	435	457	1922
Age (years)	59.1 ± 9.8	58.6 ± 9.9	57.5 ± 9.2	58.6 ± 9.7
Duration of diabetes (years)	9.2 ± 6.2	11.6 ± 6.5	8.2 ± 6.2	9.5 ± 6.3
HbA_1c_ (%)	8.2 ± 0.8	8.5 ± 0.8	8.3 ± 0.9	8.3 ± 0.8
HbA_1c_ (mmol/mol) (approx. calculated value)	66 ± 6	69 ± 6	67 ± 7	67 ± 6
Fasting plasma glucose (mmol/l)	9.7 ± 2.6	8.5 ± 2.0	9.6 ± 2.7	9.4 ± 2.5
BMI (kg/m^2^)	31.1 ± 4.7	25 ± 3.6	32.4 ± 5.4	30.0 ± 5.4

Values are means ± standard deviation (sd).

No differences were observed in the rates of diabetes-related complications or rates of co-morbid conditions (e.g. depression), which could have influenced the SF-36 outcomes.

### Glycaemic control and hypoglycaemia

HbA_1c_ in patients treated with insulin degludec was confirmed as non-inferior to insulin glargine in all three trials. Fasting plasma glucose reductions were significantly greater with insulin degludec in two trials and numerically better in one 21–23. A pre-specified meta-analysis of hypoglycaemia across the three studies showed significantly less confirmed overall (17% less) and nocturnal (36% less) hypoglycaemia, and significantly fewer severe hypoglycaemic events (86% less) for insulin degludec. Details of this hypoglycaemia meta-analysis are presented elsewhere 20. There was a weight gain of roughly 2 kg observed across the trials with both insulin degludec and insulin glargine, with no significant between-arm differences.

### Health-related quality of life—SF-36

**Figure 1:**
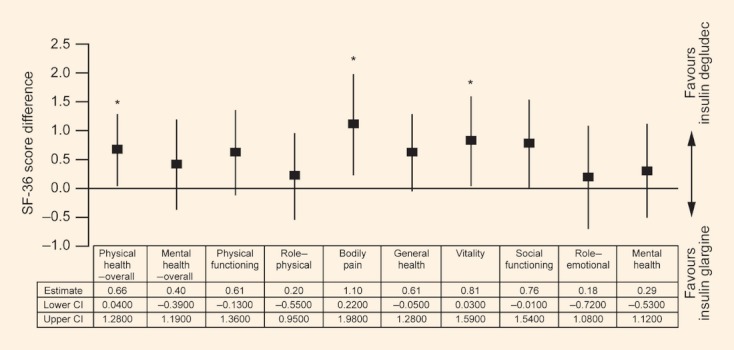
Between-treatment differences in 36-item Short Form (SF-36®) domain scores for insulin degludec vs. insulin glargine. *P < 0.05.

At end of trial, the overall physical component score was significantly higher (improved) with insulin degludec compared with insulin glargine [+0.66 (95% CI 0.04–1.28)] (Fig. 1). This was largely attributable to a difference between insulin degludec and insulin glargine of +1.10 (95% CI 0.22–1.98) in the bodily pain domain (Fig. 1). In the mental domains, vitality was significantly higher with insulin degludec vs. insulin glargine [+0.81 (95% CI 0.01–1.59)] (Fig. 1). The remaining SF-36 domains had values in favour of insulin degludec, but these were not significantly different from insulin glargine (Fig. 1).

Subjects treated with insulin degludec showed a significant improvement in all summary scores and domains, between baseline and end of trial, with the exception of the physical functioning domain (2). In the insulin glargine treatment arm, the overall physical score, role—physical and general health domains showed significant improvements between baseline and end of trial; however, the remaining summary scores and domains were unchanged (3). Between-trial heterogeneity was tested (assumed to be constant in the fixed-effects model) by specifying a model with trial as a random effect. This did not change the (point) estimates markedly, but increased the uncertainty, leaving only bodily pain with a statistically significant difference between the two treatment arms. Using the random-effects model did not improve the goodness of fit of the model according to Akaike information criterion 1974.

**Table 2:** Insulin degludec 36-item Short Form (SF-36®) within-groups scores

Type 2 diabetes basal–oral therapy	Insulin degludec									
	Baseline			End of trial			Treatment contrast			
	*n*	Least-squares mean	Standard error (se)	*n*	Least-squares mean	Standard error (se)	Contrast	95% CI, lower	95% CI, upper	*P*-value
Physical health—overall	1278	47.69	0.58	1285	48.48	0.58	0.79	0.41	1.17	**≤ 0.0001**
Mental health—overall	1278	48.73	0.69	1285	49.82	0.69	1.09	0.58	1.61	**≤ 0.0001**
Physical functioning	1269	47.65	0.63	1284	47.96	0.63	0.31	–0.15	0.76	0.1845
Role—physical	1269	47.79	0.66	1284	48.55	0.66	0.76	0.26	1.26	**0.0027**
Bodily pain	1270	49.55	0.71	1284	50.53	0.71	0.98	0.41	1.54	**0.0007**
General health	1271	44.60	0.63	1283	46.31	0.63	1.71	1.29	2.13	**< 0.0001**
Vitality	1266	50.83	0.67	1285	52.28	0.67	1.45	0.96	1.95	**< 0.0001**
Social functioning	1277	48.59	0.63	1285	49.36	0.63	0.77	0.26	1.27	**0.0031**
Role—emotional	1266	46.70	0.74	1284	47.70	0.74	1.01	0.41	1.61	**0.0010**
Mental health	1265	48.76	0.70	1285	49.60	0.70	0.84	0.31	1.37	**0.0018**

Bold values indicate statistical significance.

**Table 3:** Insulin glargine 36-item Short Form (SF-36®) within-groups scores

Type 2 diabetes basal–oral therapy	Insulin glargine									
	Baseline			End of trial			Treatment contrast			
*n*	Least-squares mean	Standard error (se)	*n*	Least-squares mean	Standard error (se)	Contrast	95% CI, lower	95% CI, upper	*P*-value
Physical health—overall	623	46.51	0.57	629	47.06	0.57	0.55	0.03	1.08	**0.0376**
Mental health—overall	623	48.95	0.66	629	49.40	0.66	0.46	–0.24	1.16	0.1976
Physical functioning	619	46.33	0.66	629	46.58	0.66	0.25	–0.40	0.91	0.4434
Role—physical	617	47.13	0.63	628	47.79	0.63	0.66	0.04	1.28	**0.0363**
Bodily pain	616	48.89	0.71	629	49.05	0.70	0.16	–0.59	0.91	0.6741
General health	618	43.54	0.61	628	44.90	0.61	1.36	0.78	1.94	**< 0.0001**
Vitality	617	50.67	0.64	629	51.26	0.64	0.59	–0.12	1.30	0.1055
Social functioning	622	48.14	0.64	629	48.31	0.64	0.17	–0.54	0.88	0.6379
Role—emotional	614	46.60	0.71	627	47.16	0.70	0.56	–0.24	1.35	0.1706
Mental health	617	48.57	0.68	629	49.11	0.68	0.54	–0.17	1.25	0.1387

Bold values indicate statistical significance.

## Discussion

This meta-analysis demonstrates that treatment with insulin degludec leads to an improvement in health-related quality of life compared with insulin glargine in patients with Type 2 diabetes starting on insulin therapy. Specifically, patients treated with insulin degludec reported significantly less bodily pain, significantly better vitality and significantly better overall physical health at the end of the studies, compared with insulin glargine. The present study confirms the findings of recently published trials, in both Type 1 diabetes 17 and Type 2 diabetes basal–bolus therapy 16, which demonstrated an improvement in health-related quality of life with insulin degludec vs. insulin glargine. As previously stated, hypoglycaemic events are a major contributor to reduced health-related quality of life, affecting both mental and physical health in patients with diabetes. Therefore, an explanation for the difference in health-related quality of life domains between insulin degludec and insulin glargine may lie with the decreased rates of hypoglycaemia observed for insulin degludec in head-to-head trials with insulin glargine—although further research is needed to confirm this hypothesis. It is also conceivable that the flat profile of insulin degludec generates an improved sense of well-being, possibly as a result of fewer near-hypoglycaemic events, which is traceable in the SF-36, but not in the glycaemic parameters; or by increasing the patient's confidence in the predictability and effectiveness of insulin degludec. The reduced variability in blood glucose levels exhibited by insulin degludec may also improve the utilization of metabolic fuel substrates in the periphery, leading to increased vitality and physical functioning. In seeking to account for the reduction in bodily pain experienced with insulin degludec treatment, it should be noted that there is a difference in the preparation of the insulins, with insulin glargine being acidic, having a pH of 4, and insulin degludec being pH neutral 26. Although there was no difference in the number of injection-site reactions in the studies comprising our analysis, this does not rule out the possibility of a difference in post-injection discomfort experienced between treatments.

The clinical trials comprising this meta-analysis are some of the first to measure health-related quality of life directly in patients with Type 2 diabetes. Previous studies have used disease-specific measures, but not generic health-related quality of life, perhaps because of the perceived difficulty in observing differences in quality-of-life domains 27. The SF-36 does not have an established minimal important difference in diabetes; however, the SF-36 version 2 user manual suggests that even low scores (> 1) are relevant in other chronic diseases. For example, having an allergy reduces the SF-36 scores by 0.1 to 0.8 points 10.

Disease-specific treatment satisfaction questionnaires were also applied in degludec trials, including the studies in the meta-analysis. In general, while the scores numerically favoured insulin degludec, there were no significant differences between treatments. This may support the use of a generic measure (SF-36), which has greater latitude in evaluating broader health-related quality of life issues. Using a symptom-specific scale (e.g. the hypoglycaemia fear scale) may have provided useful information on impact of hypoglycaemia on health-related quality of life in this analysis. However, whilst we recognize the importance of hypoglycaemia in health-related quality of life assessment, there are many contributing factors to health-related quality of life and a broad approach may be more likely to elicit this information. Similarly, alternative generic measures, such as the EuroQoL-5D, provide useful information for health economic analysis by yielding a single value for health utility. The EuroQoL-5D is known to lack sensitivity in subjects whose quality of life is in the range expected for these studies and, while important, we consider that it would be appropriate for health utility analysis to be conducted as a separate investigation.

Other studies evaluating insulin analogues compared with human insulins have shown a benefit to treatment satisfaction: investigators have suggested that this may be linked with the lower variability in plasma glucose concentrations and reduced risk of hypoglycaemia associated with insulin analogues 28. Although there have been clinical trials evaluating health-related quality of life with rapid-acting insulin analogues, ours is one of the first to directly assess health-related quality of life in a basal insulin. A recent Cochrane review of the long-acting insulin class showed that the rate of symptomatic, overall and nocturnal hypoglycaemia was statistically significantly lower in patients treated with either insulin glargine or insulin detemir compared with neutral protamine Hagedorn (NPH) insulin. However, no evidence for a beneficial health-related quality of life effect could be obtained as none of the included trials reported health-related quality of life 29. The difference in health-related quality of life observed between two basal insulins in our meta-analysis supports the inclusion of patient-reported outcomes when assessing efficacy in clinical trials.

Open-label trials are often considered prone to bias; however, the duration of the studies in this meta-analysis, 26 or 52 weeks, was considered sufficient to ensure that any expectations relating to the initiation of insulin therapy would have ‘washed out’ by the end of the trial. It is not clear which direction such a bias would act in, as subjects may be affected either by the availability of a novel therapy or by the counselling associated with being treated with an investigational medical product. In addition, baseline values should have been unaffected by brand-specific bias because the study population was insulin naive and completion of the SF-36 questionnaire happened prior to randomization. Moreover, empirical estimates of open-label vs. double-blind trials suggest that bias effects, while real, are modest 30. The trials used in this meta-analysis were well designed and carefully implemented to minimize bias and, as a result, differential loss to follow-up was very low. Despite these safeguards, we cannot exclude a psychological bias between the patient groups, especially given the increasing trend for patients to self-research their condition and treatment on the Internet. One criticism of the SF-36 questionnaire has been the lack of a sleep variable. Sleep—a component of vitality—is often disturbed in patients with diabetes, particularly in individuals experiencing high rates of nocturnal hypoglycaemia, and, given that insulin degludec has shown a reduced rate of nocturnal hypoglycaemia compared with insulin glargine, the present study may underestimate this component of health-related quality of life with insulin degludec, as it did not specifically isolate the overnight period in the evaluation 14,20.

The strengths of this meta-analysis include the prospective nature of the study; the use of high-quality, patient-level data from regulatory trials; the randomized, controlled trial context; and the selection of an insulin-naive population with reduced expectations of starting insulin therapy. The randomized allocation of subjects means that the distribution of known and unknown confounders should, by chance, be approximately equally distributed between treatment groups. Another major strength of the present analysis is that a widely used and thoroughly validated health-related quality of life instrument (SF-36 version 2) was applied across several trials, enabling a consistent evaluation of health-related quality of life. Further analysis should be conducted to establish whether the observed advantage of insulin degludec, in terms of health status, translates into an improvement in health utility. This is a step beyond the SF-36 design, but would aid healthcare providers in assessing the economic effectiveness of insulin degludec compared with currently available diabetes treatments.

In conclusion, it is acknowledged by healthcare professionals that diabetes has a serious, negative impact on health-related quality of life. The results of this pre-planned meta-analysis, utilizing patient-level data, demonstrate that insulin degludec significantly improves health-related quality of life compared with insulin glargine, in patients with Type 2 diabetes starting insulin therapy.

## Funding sources

The clinical trials in this meta-analysis were sponsored by Novo Nordisk A/S (Bagsværd, Denmark).

## Competing interests

NF has received funding for research, consulting and travel from Novo Nordisk, Eli Lilly, Sanofi and Medtronic. LM has acted as consultant and served on advisory boards for Novo Nordisk and Sanofi Diabetes. He has also received research support from MannKind, Pfizer and Boehringer Ingelheim, and has shares in Dexcom. TC and MLW are employed by, and shareholders in, Novo Nordisk A/S, Denmark. JJ has served on advisory boards for Boehringer Ingelheim, Eli Lilly & Co., GSK, Novo Nordisk A/S and Pfizer. He has also received speaker fees from Aidera, Astra Zeneca, Eli Lilly & Co., GSK, MSD, Novo Nordisk, Pfizer and Roche. RR received research support from Sanofi and Novo Nordisk and served on advisory boards for both companies.

## Supporting Information

Figure S1 SF-36 version 2 health survey.
