# Supplementary material for: Insulin degludec improves health-related quality of life (SF-36®) compared with insulin glargine in people with Type 2 diabetes starting on basal insulin: a meta-analysis of phase 3a trials
Source: Diabet Med. 2013 Jan 21;30(2):226–32. doi: 10.1111/dme.12086 (PMC3579236; doi:10.1111/dme.12086)
Supplement: Figure S1 — SF-36 version 2 health survey. [file dme0030-0226-sd1.docx]

Supplemental Fig. S1: SF-36® version 2 health survey
